# Autapses enable temporal pattern recognition in spiking neural networks

**DOI:** 10.1371/journal.pone.0339918

**Published:** 2026-02-02

**Authors:** Muhammad Yaqoob, Volker Steuber, Borys Wróbel

**Affiliations:** 1 Department of Computer Science, University of Hertfordshire, Hatfield, United Kingdom; 2 Laboratory of Bioengineering, Institute of Molecular Biology and Biotechnology, Adam Mickiewicz University in Poznań, Poland; 3 European Institute for Brain Research, Amstelveen, The Netherlands; 4 Nectome Inc., Vancouver, Washington, United States; Lanzhou University of Technology, CHINA

## Abstract

Most sensory stimuli are temporal in structure. How action potentials encode the information incoming from sensory stimuli remains one of the central research questions in neuroscience. Precise spike timing is known to represent information in spiking neuronal networks, yet the information processing mechanisms of spiking neuronal networks is poorly understood. One feasible way to understand the processing mechanism of a spiking network is to associate the structural connectivity of the network with the corresponding functional behaviour. This work demonstrates the structure-function mapping of spiking networks evolved (or handcrafted) for a temporal pattern recognition task. The task is to recognise a specific order of the input signals so that the output neuron of the network spikes only for the correct placement and remains silent for all others. The minimal networks obtained for this task revealed two complementary roles of autapses in recognition. First, autapses enable a seamless transition to the next network state when a new input signal arrives. Second, in the absence of the input signal, they allow the network to maintain a network state for an extended period, a form of memory. To show that the recognition task is accomplished by transitions between network states, we map the network states of a functional spiking neural network (SNN) onto the states of a finite-state transducer (FST). Finally, based on our understanding, we define rules for constructing the topology of a network handcrafted for recognising a subsequence of signals in a particular order. The analysis of minimal networks recognising patterns of different lengths revealed a positive correlation between the pattern length and the number of autaptic connections in the network. Furthermore, in agreement with the behaviour of neurons in the network, we were able to associate specific functional roles of ’locking,’ ’switching,’ and ’accepting’ to neurons.

## Introduction

In biological neural networks, the problem of temporal pattern recognition refers to identifying a sequence of input signals or spikes that carry information. The brain processes such sequences elegantly, responds quickly [1–3], and can efficiently differentiate spike patterns distributed across time and space [4]. However, little is known about how individual neurons contribute to processing temporal signals. Interpreting a sequence of spikes generated by a neuron (or a group of neurons) to determine the spatial or temporal structure of a stimulus is a fundamental problem in neuroscience [5]. Analogue sensory information from different modalities, including olfactory, auditory, and visual input, is encoded in the form of spikes [6], and processed precisely by the brain with incredible speed [7]. The timing of the spikes captures the varying transient intensities of the stimulus, and even a single spike may represent important information after the stimulus onset [8,9]. These results suggest that temporal features of spikes can precisely represent a stimulus and convey information in biological and artificial spiking neural networks [10]. In the past decades, it has been established that sensory information is represented by the precise timing of spikes in the somatosensory, auditory, visual, and olfactory systems [4,9,11–16]. Furthermore, several studies have shown that information processing in the nervous system is linked to transitions from one spiking behaviour to another [5,17–22]. However, despite extensive research, the association between structural connectivity and the functional behaviour of neural systems remains unclear. Understanding how spiking neural networks process information to gain insight into the computational capabilities of a biological brain is one of the most challenging problems in computational neuroscience [23,24]. A possible way to study the complex computational processing capabilities of the brain is to artificially produce networks with biologically plausible neurons that can perform a computational task.

From a computational perspective, processing temporal spike patterns is a general computational task performed by the brain [25,26]. There are two common ways of learning to recognise temporal patterns: (i) adjusting conduction delays [27–30], (ii) selecting conduction delays from a spectrum of existing delays [19,31,32]. Spiking neural networks (SNNs) are documented to differentiate temporal patterns by exploiting different time delays and pathways in the network [6]. In neural systems, time delays can be adjusted at the level of synapse, axon, or soma. Adjusting these delays at one or more levels in the network can uncover features of a signal [33]. On the other hand, it is possible that signals produced at different timings arrive together at the readout neuron, generating a maximal response. This phenomenon is used for edge detection in the visual system [34]. Moreover, delays in the network can also be used to identify keywords in continuous speech [35]. This work provides a new way of learning to recognise temporal patterns by evolving (with a method inspired by natural evolution) the topology and connection weights of a population of SNNs, without changing or selecting conduction delays.

Artificial spiking neural networks (SNNs) can process a large amount of data using an efficient spiking communication mechanism between neurons such that information is transmitted only when a neuron spikes [9]. Due to their similarities to biological networks, SNNs are widely used to model information processing in animal brains. SNNs have been shown to be computationally more powerful, especially in terms of speed and accuracy, and can solve problems more efficiently than non-spiking neural networks [36,37]. Furthermore, SNNs are considered robust to noise and damage, and their functionality gradually deteriorates when confronted with perturbations [38,39]. To understand the learning and information processing mechanism of SNNs, we first need to obtain networks that can perform a specific computational task. Considering the fact that information received from the majority of sensory modalities is temporal in structure, in this work we evolved the structural topology and connection weights of a population of SNNs using a genetic algorithm to recognise temporal patterns. It is documented that temporal patterns can be detected by recurrent connections (a form of memory) or delays in the network [28,40]. In general, a recurrent connection can be of any size. However, the shortest possible recurrent connection observed in the nervous system is a self-connection. Self-connections (autaptic connections or autapses) are recurrent synaptic connections between the axon and dendrites or soma of a single neuron (either excitatory or inhibitory). Discovered five decades ago [41], autapses can be observed in the mammalian brain in the neocortex, hippocampus, and cerebellum [42]. Recent studies suggest possible roles of autapses in the synchronisation of networks [43], flexible working memory networks [44], and coherence resonance [45].

It has been demonstrated that a single autaptic-neuron (a neuron with a self-loop) can promote the detection of weak signals through stochastic resonance in a Hodgkin-Huxley neuron model [46]. Based on the Hindmarsh–Rose (HR) neuron model [47], a recent study proposed a memristive autapse-coupled neuron model (MACNM) that can exhibit a variety of complex firing dynamics under external electromagnetic radiation [48]. These studies highlight the importance of autapses in information processing in neural networks. Previously, autapses have been relatively under-explored and were sometimes regarded as wiring errors in the nervous system [43].

Models of small networks may reveal many interesting properties in neuroscience and could aid our understanding of how neuronal networks function in the animal brain [49,50]. They have been studied in various artificial settings, including time-continuous and time-discrete models, as well as networks composed of spiking or rate-based neurons. [49,51–53]. It has been documented that small neural networks can represent the mechanism driving neural activity in a biological circuit [51,54,55]. Other studies have analysed the dynamics of small neural networks with respect to bifurcation and stability. They observed that changes in parameters can lead to transitions between different network states or activity patterns. [51,55–57]. Previously, we demonstrated that small spiking neural networks can be evolved to recognise a pattern of three signals in the presence of noise and perturbation. We also reported that the network activity can be mapped to a deterministic automaton to show that the pattern recognition happens with a deterministic transition from one state to another [39,58,59].

Moreover, small spiking neural networks could help in estimating network connectivity from network observations, a challenging problem in neuroscience [60]. The inferred network structure could help in determining how brain areas are connected to study both normal and abnormal brain function. In this regard, analytical methods have been proposed to determine all set of networks, via coupling strengths, that produce a given pattern dynamics [61,62]. In [63], a model-invariant theory is suggested to reconstruct network connectivity using event timing patterns from spiking activity. The inferred network could reliably determine the presence or absence of interactions, their nature (excitaory or inhibitory) and produce both regular and irregular patterns. Another recent study inferred synaptic weights by analysing firing rates in variable time bins within a heterogeneous feed-forward network of excitatory, inhibitory, and unconnected neurons [64].

The computational task for SNNs in this work is to recognise a given subsequence of signals in a continuous stream of input signals. The network consisted of a number of input channels (equal to the number of distinct input signals), a layer of interneurons (neurons in the hidden layer), and an output neuron. The *Output* neuron is expected to spike for the correct input pattern while remaining silent for other input patterns. We show the importance of autapses by revealing their possible functional role in state maintenance—a form of memory in the network. Furthermore, SNNs with autaptic connections tend to evolve a simplified switching mechanism to recognise patterns of lengths three and four [59]. Consistent with the evolved networks, we define rules for constructing the topology of a network by hand to recognise patterns up to length six with six interneurons, demonstrating a perfect linear relation between the number of signals in the pattern and the number of interneurons in the network. We show that autapses are crucial for switching the network between states and for maintaining a network state. Furthermore, our results indicate that the pattern length to be recognised correlates positively with the number of autaptic connections in the network. More specifically, recognising a pattern of *n* signals requires a network of *n* interneurons with *n*–1 autaptic connections. Finally, we demonstrate that, in addition to the other interneurons, a successful recogniser network must have three specialised neurons: a *Lock*, a *Switch*, and an *Accept* neuron. The activity of *Lock* prevents *Output* from spiking, except when the network receives the second-to-last correct input signal and allows the *Output* neuron to spike in response to the correct last input. The *Switch* neuron is responsible for transitions between the network start state and inter-signal network states. The *Accept* neuron forces spike(s) in the *Output* neuron if the lock is released by the penultimate signal in the pattern to be recognised and switches the network back to the *Start* state by activating the *Switch* neuron.

## Methods

The SNNs in this work consist of adaptive exponential integrate and fire neurons (AdEx) [57]. Each AdEx neuron has four state variables: membrane potential *V*, excitatory conductance *g*_*ex*_, inhibitory conductance *g*_*in*_, and adaptation *w*, along with 14 parameters (Table 1); all neurons in the network have the same parameter values.

**Table 1:** AdEx parameters for tonic spiking used in this work.

Parameter		Value
*E*_*l*_	effective rest potential	-70 mV
*E*_*in*_	inhibitory reversal potential	-70 mV
*E*_*ex*_	excitatory reversal potential	0 mV
$V_{r}$	reset voltage	-58 mV
$V_{T}$	effective threshold potential	-50mV
$V_{th}$	spike detection threshold	0 mV
$\Delta_{T}$	threshold slope factor	2 mV
*C*	total capacitance	0.2 nF
*g*_*l*_	total leak conductance	10 nS
*a*	adaptation conductance	2 nS
*b*	spike-triggered adaptation	0 pA
$\tau_{w}$	adaptation time constant	30 ms
$\tau_{ex}$	time constant for the excitatory conductance	5 ms
$\tau_{in}$	time constant for the inhibitory conductance	5 ms

Out of the 14 parameters, four bifurcation parameters are responsible for the spiking behaviour: the adaptation conductance *a*, the spike-triggered adaptation *b*, the adaptation time constant $\tau_{w}$, and the resting potential $V_{r}$. The remaining scaling parameters are: the total capacitance *C*, the total leak conductance *g*_*l*_, the effective rest potential *E*_*l*_, the inhibitory *E*_*in*_ and the excitatory *E*_*ex*_ reversal potential, the threshold slope factor $\Delta_{T}$, the effective threshold potential $V_{T}$, and two additional time constants, $\tau_{ex}$ (for the excitatory conductance) and $\tau_{in}$ (for the inhibitory conductance).

$$C\frac{dV}{dt}=g_{ex}(E_{ex}-V)+g_{in}(E_{in}-V)-w+g_{l}(E_{l}-V+\Delta_{T}e^{\frac{V-V_{T}}{\Delta_{T}}})$$ (1)

$$\tau_{w}\frac{dw}{dt}=a(V-E_{l})-w$$ (2)

$$\frac{dg_{ex}}{dt}=\frac{-g_{ex}}{\tau_{ex}}$$ (3)

$$\frac{dg_{in}}{dt}=\frac{-g_{in}}{\tau_{in}}$$ (4)

The exponential term in equation 1 defines the spike generation mechanism and the ascent of the action potential. In the mathematical description of the model, a spike is detected at time *t^f^* when the membrane potential crosses an arbitrary firing threshold value (larger than $V_{T}$, here 0 mV). When this happens, the integration of the differential equations (Equations 1 to 4) is stopped, the spike time *t^f^* is recorded, and the voltage is reset to a fixed value $V_{r}$. This reset describes the descent of the action potential, given by:


$$\text{at}\;t=t^{f}\;\text{reset}\;V⟶V_{r}$$

Simultaneously, when a spike is recorded at time *t^f^*, the adaptation current *w* increases by an amount *b*:


$$\text{at}\;t=t^{f}\;\text{reset}\;w⟶w+b$$

In the AdEx model, synaptic connections are modelled as abstract excitatory and inhibitory conductances that influence the membrane potential rather than using a detailed biophysical synapse model. Moreover, autapses are implemented in the same way as other synaptic connections; the only difference is that they connect the neuron to itself, and the autaptic current follows the same computational formulation as any other synaptic current.

The interaction between the differential equations of the AdEx model and the above two discrete resets can generate a variety of spiking behaviours [57]. In this work, we use the parameters (Table 1) for producing tonic spiking when a constant step current is injected into a neuron. The state variables are integrated with a time step of 1 ms using Euler integration (using a time of 0.1 ms or more precise integration algorithms like Runge-Kutta did not affect the results in preliminary experiments).

In addition, noise was modelled by adding a random perturbation, drawn from a normal (Gaussian) distribution N[mean=0, SD=1], to the membrane potential at each network step. The added zero-mean Gaussian noise simulates stochastic fluctuations in the membrane potential, similar to synaptic or channel noise observed in biological neurons.

### Evolution of networks for recognising a pattern of length 3

**Fig 1:**
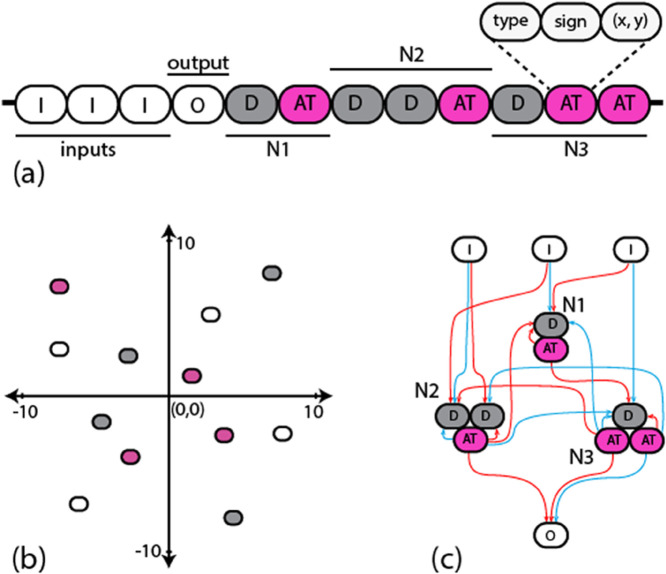
(a) The structure of a random linear genome in the population, encoding a network with three interneurons, three input nodes and one Output neuron. A sequence of D elements (dendrites) followed by a sequence of AT elements (axon terminals) is considered one neuron. Each element has a type (I, O, D, AT), a sign (+, -) and (x, y) coordinates in 2D space. The strength of a connection between two elements is defined as an inverse function of the Euclidean distance between their coordinates. (b) Shows genetic elements (I, O, D, AT) in 2D space. (c) Network Topology of the obtained spiking neural network. Excitatory (inhibitory) connections are represented by red (blue) color.

To obtain networks that recognise a pattern of three signals in a particular order, we used a genetic algorithm originally developed for evolving gene regulatory networks [65,66], where the topologies of the networks in the population are encoded as linear genomes. Each genome contains a list of genetic elements such that each element has three attributes: a type (input I, output O, dendrite D, or axon terminal AT), a sign (+, -), and coordinates (x, y) (Fig 1). A sequence of D elements followed by a sequence of AT elements encodes one neuron in the network. To determine the total synaptic strength (weight) of a connection between two neurons in the network, we aggregate the affinity between all AT elements of the presynaptic neuron and all D elements of the postsynaptic neuron. Each element has (x,y) coordinates in an abstract affinity space; the smaller the Euclidean distance between two elements i and j, the larger the contribution to the synaptic strength given by $s_{i}s_{j}\frac{2(5-d_{i,j})}{10d_{i,j}+1}$. If the signs ($s_{i}\;and\;s_{j}$) are the same (different), the contribution is positive (negative). The pre-post connection is established only if the absolute value of the sum of the weight contributions is above a cut-off threshold (to prevent full connectivity); a positive (negative) sum results in an excitatory (inhibitory) connection. In this work, a given presynaptic neuron can excite some of its targets and inhibit others – violating Dale’s principle [67]. However, any violating neuron in the evolved network could be transformed to follow Dale’s principle by dividing it into two parts, one excitatory and one inhibitory (each having the same number of incoming connections, with the same weights, as the original neuron), such that the network’s performance is not compromised [59]. In this work, we do not perform this transformation in the analysis of the networks for simplicity.

We use the genetic algorithm [65] with a constant population size of 300. The initial population is created with random topologies encoded as linear genomes. Subsequent generations are created with a size two tournament selection and an elite count of 10 individuals. Four genetic operators, (point mutation, deletion, duplication, and crossover) were fine-tuned for faster convergence [39]. If an element is chosen for a point mutation (with a probability of 0.1 per element), its (x,y) coordinates are changed in a random direction by a distance drawn from a normal distribution N[mean=0, SD=1]. Deletions occurred with a probability of 0.0005 per genome. The starting point of the deletion is chosen randomly with all elements being equally likely to be selected. The length of the deleted segment is determined using a geometric distribution with an average length of 11. The duplications happen with a probability of 0.001 per genome which is twice as likely as deletions. The starting point for duplication and the site where the duplicated segment is inserted are chosen randomly, with all elements being equally likely to be selected. The length of the duplicated segment follows a geometric distribution with an average length of 11. For multi-point crossover, two parent genomes A and B are chosen from the population with a binary tournament selection. A cursor is set at the first genetic element of both parent genomes to begin the crossover process. Then one of the four actions is chosen: (i) Copy an element from parent A to the offspring and move the cursor on both genomes. (ii) Copy an element from parent B to the offspring and move the cursor on both genomes. (iii) Copy an element from parent A to the offspring and move the cursor only for parent A. (iv) Copy an element from parent B to the offspring and move the cursor only for parent B. Actions (i) and (ii) are equally likely with a probability of 0.12 and actions (iii) and (iv) are also equally likely with a probability of 0.003. After an element is copied using one of the four actions, there is a 70% chance that the same action will be repeated and a 30% chance that a new action will be chosen with the same probability ratios as above.

A recurrent SNN for recognising a pattern of three signals is found to require at least three interneurons, one *Output* neuron and three input nodes. The inputs are not allowed to connect to *Output* directly, and only the interneurons are allowed to have self-loops (autaptic connections). During evolution, the genetic algorithm can add/remove connections by moving the (x,y) coordinates associated with each genetic element.

The task of the networks is to recognise a pattern of three signals in a continuous stream of signals in which all signals (A, B, and C) occur with equal probability. In an input sequence, the length (duration) of a signal is 6 ms, followed by a silence interval of 24 ms. During evolution, each network in the population is evaluated for six sequences of signals. Four out of these six sequences are generated randomly with an equiprobable occurrence of the three signals (A, B, and C). The remaining two sequences are created by concatenating hard-to-differentiate patterns (ABA, ABB, ABC, BBC) in random order.

The fitness function rewards the spiking of the *Output* neuron in the correct inter-stimulus interval after the occurrence of the correct pattern ABC and penalises spikes in all other intervals:

$$f_{fitness}=1-(R-kP)$$ (5)

where *R* is the normalised reward given by the number of inter-stimulus intervals after C in which the *Output* neuron spikes after receiving the correct pattern ABC divided by the total number of correct patterns in the input sequence. The inter-stimulus interval is defined as the interval between the onsets of two consecutive stimuli. The penalty *P* is the number of inter-stimulus intervals in which the *Output* neuron spikes incorrectly, divided by the total number of inter-stimulus intervals in the sequence (which is one less than the total number of signals in the sequence). As this denominator is large, the normalised penalty *P* is amplified by a constant *k* = 4.

Although the weight cut-off threshold is in place to prevent full network connectivity, the evolutionary algorithm tends to produce superfluous connections that can be pruned without impairing the performance of the network [59]. To aid the network analysis, we prune the evolved networks by removing a random connection and testing if the performance of the network is compromised. If it is, the connection is put back and labelled as vital. Otherwise, the excessive connection is removed from the network. This process is repeated until only vital connections remain in the network.

Pruning revealed structural similarities between the networks obtained from different independent evolutionary runs; thus, the networks that recognised a pattern of three signals were either equal or isomorphic. Moreover, it stood out that the recognition of a pattern depends on the transitions between the network states. Therefore, the states of an evolved network can be mapped onto the states of the corresponding finite state transducer (FST), a formal model of computation – accepting a string of three letters.

### Handcrafting networks for recognising patterns of length four and above

**Fig 2:**
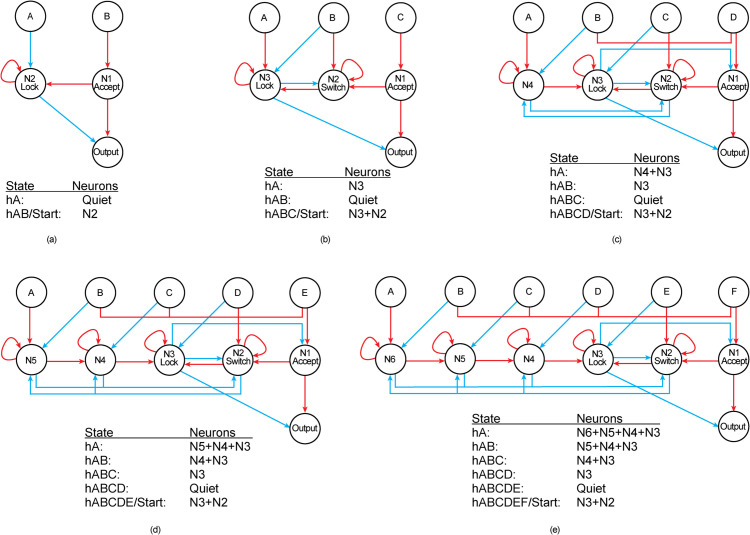
Networks recognising patterns of lengths 2 to 6. The first two networks in panels a and b were obtained with artificial evolution. The blue (red) arrows represent inhibitory (excitatory) connections. The table below each network shows transitions between network states, represented by the number of active neurons. The topology is extended by hand to recognise patterns of lengths 4, 5, and 6 (c-e).

Understanding the working mechanism of three-signal networks allowed handcrafting network topologies that recognise longer patterns. For example, a network topology recognising a pattern of three signals can be extended so that it recognises a pattern of four signals by adding a new input, an interneuron and six synaptic connections (Fig 2b-c). In the extended network, the input stimuli are renamed ABCD. The newly added input A and the neuron *N*4 connect to the existing network; input A connects to *N*4 with an excitatory connection, input B (previously named input A and connected to neuron *N*3) excites *N*2 (the *Switch* neuron), and input B also connects to the newly added neuron *N*4 with an inhibitory connection. The neuron *N*4 connects to *N*3 and to itself with excitatory connections, and the neuron *N*4 and *N*2 (the *Switch*) inhibit each other and thus are mutually exclusive in terms of their activation state. In networks recognising longer patterns, two more connections are essential for seamless switching back to the start state after receiving the last input signal. Therefore, these connections are introduced in networks recognising patterns of length four and above: (i) the last input excites the *Switch* neuron, and (ii) the *Lock* (*N*3) neuron inhibits the *Accept* (*N*1) neuron. These general rules can be used to extend the topology of a four-signal network to obtain the topology for recognising patterns of lengths five and six (Fig 2d-e).

The structural topology and the sign of the connections are fixed in the handcrafted networks. However, the weights need to be optimised for efficient recognition. We use a genetic algorithm to optimise only the weights of connections. Here, an individual’s genome is the adjacency list of the handcrafted network. In the initial generation, a population of 100 individuals sharing the same structural topology is created. The weights of the excitatory (inhibitory) connections are drawn from a uniform distribution U[0, 10] (U[-10, 0]). The population size is kept constant during evolution with 10 individuals in the elite. The only mutation operator is adding a random number drawn from a normal distribution N[mean=0, SD=1] to the connection weight chosen with a probability of 0.1. Subsequent generations are created with a size two tournament selection, and the fitness function is identical to the one for evolving both the topology and the connection weights (Equation 5), rewarding spikes in the correct inter-stimulus interval and penalising spikes elsewhere.

The number of possible patterns increases exponentially with the length of the pattern. There are *n^n^* possible orderings for *n* signals. For example, a pattern of four signals has 4^4^ = 256 (AAAA to DDDD) possible permutations, and a pattern of six signals has 6^6^ = 46656 (AAAAAA to FFFFFF). This means that when *n* is large, a given permutation will occur less frequently in a random input stream of signals, and some patterns may not occur at all (so the networks cannot be penalised for recognising them). We have therefore designed a genetic algorithm that runs in two stages. Consider evolving networks for recognising a pattern of length four in a given order. In the first stage, the networks are optimised only for patterns similar to the target pattern ABCD. These similar patterns have the form AXXX, XXXD, where XXX in AXXX (XXXD) is replaced by all 27 possible patterns of BCD (ABC). A sequence of 10,000 signals is created by randomly concatenating the 54 patterns (ABBB to ADDD, AAAD to CCCD). Once the genetic algorithm converges for the first one (finds a network that only responds to the correct pattern ABCD and remains silent for all other patterns), the second stage begins by identifying hard-to-recognise patterns. This requires evaluating the network for a large random sequence with an equiprobable occurrence of signals A, B, C and D. A pattern is considered hard if the network responds incorrectly to multiple occurrences (at least 40% of the total number of occurrences) of a given pattern in a random sequence. Evolution continues with the fitness function in which the penalty coefficient is equal to 50, and the input sequence of 10,000 signals is created by randomly concatenating the 54 patterns (AXXX patterns with XXXD patterns) and hard patterns with equal probability. The second stage (identification of hard patterns, evolution) repeats until the algorithm converges and no hard patterns remain.

## Results

### States of the network correspond to the states of a finite state transducer

**Fig 3:**
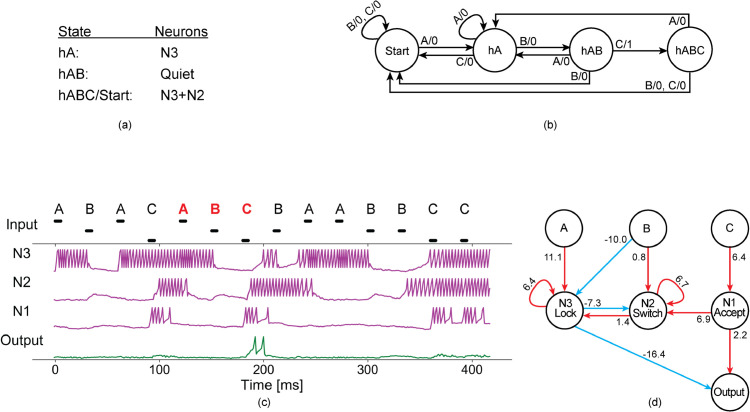
The working mechanism of a network evolved in the presence of membrane potential noise. (a) The states of the network and the corresponding activity of the neurons in the pruned network. (b) The minimal finite-state transducer that recognises ABC. (c) The activity of the network when a random stream of input signals A, B, and C is received. The different input levels represent input being received from different input channels; the voltage remains the same for all inputs. (d) The topology of the pruned evolved network.

To understand how the evolved networks work, we show that the activity of a network can be mapped onto the states of a finite state transducer (FST) [38,59]. An FST is a finite state machine generally used for analysing time-structured data [68]. For example, an FST that accepts a string of three letters needs to have four distinct states (Fig 3b), and so does an SNN evolved to recognise a pattern of three signals (Fig 3a). We established an association between the network states of a pruned network and the states of the corresponding minimal FST. This association can also be obtained for an evolved network, but the analysis of the network is more difficult [59,69]. The *Start* state of the network corresponds to the continuous spiking of the neurons *N*3 and *N*2 (Fig 3c). Once the network receives the first correct signal A, it goes into the *hA* (for “had A”) state, maintained by continuous spiking of the *N*3 neuron only. The presence of an excitatory loop (autapse) on *N*3 preserves the network state until the next signal (A, B, or C) is received. If the network receives another A, it remains in the *hA* state. However, if the network receives a B, it transforms to the *hAB* state retained by no activity in the network (all neurons in the network are quiescent). If C rather than B arrives after A, the network goes back to the *Start* state from the *hA* state.

Suppose that the network is in the *hAB* state and a signal C arrives. As a result, the *Accept* neuron spikes several times and, in turn, activates the *Output* neuron. The *Accept* neuron also activates the *Switch* neuron to transform the network back into the *Start* state. On the other hand, receiving a signal B when the network is in the *hAB* state activates the neuron *N*2 (explained by the weak excitatory connection from input B to *N*2), which in turn activates *N*3, transforming the network into the *Start* state, represented by continuous spiking of *N*2 and *N*3. Furthermore, if the network receives an A while in the *hAB* state, the network switches back to the *hA* state, characterised by continuous spiking of *N*3, thanks to the excitatory autapse *N*3.

### Specialised role of neurons in the network and the importance of autapses

The pruned networks obtained from independent evolutionary runs for recognising a pattern of three signals were equal or isomorphic [38,59], allowing us to discover their working mechanism. All resulting networks accomplished the task with four neurons (three interneurons and one *Output* neuron) and 11 connections (three inhibitory and eight excitatory). Furthermore, networks capable of maintaining network states exhibited self-excitatory loops (excitatory autapses) on at least two interneurons [38,59]. To describe the working mechanism of the networks, first, the role of excitatory autapses is identified. Then, specific roles are associated with the interneurons on the basis of their spiking behaviour. Finally, we demonstrate the contribution of each connection to pattern recognition. The existence of excitatory autapses enables the evolved networks to maintain network states irrespective of the silent intervals between signals. These recurrent connections permit three active states: the *hA* state by persistent spiking of the *Lock* neuron, and the *hABC*/*Start* states by causing tonic spiking of the *Lock* and *Switch* neurons (Fig 3a). The only difference between the *Start* and the *hABC* state is the intermittent spiking of the *Output* neuron. During the evolutionary process, both the *Lock* and the *Switch* neurons formed excitatory autapses with sufficient weights to prevent spiking activity from dying out in the absence of input activity (Fig 3d). Hence, the ability of the network to maintain a state for longer silent intervals requires the formation of autapses in these minimal networks [39,59].

The interneurons have specialised roles of *locking*, *switching* and *accepting*. Self-excitation of the *Lock* neuron prevents the *Output* neuron from spiking, except when the second-to-last correct input signal (for example, B in ABC) shuts down the *Lock* neuron, enabling the *Output* neuron to spike for the last correct input signal (C in ABC). If the penultimate correct input signal releases the *Lock*, the *Accept* neuron activates the *Output* neuron on receiving the last correct input. The *Accept* neuron also sends a signal to the *Switch* neuron, transforming the network back to the *Start* state. The *Switch* neuron is responsible for the transitions between the *Start* state (when *Switch* is active) and the intermediate states *hA* and *hAB* (when *Switch* is quiescent).

### Contribution of each connection to pattern recognition

The contribution of each synaptic connection to the recognition of input patterns is determined by its effect on the behaviour of postsynaptic neurons. In Fig 3, the excitatory connection from the input A to the neuron *N*3 (A→N3) activates *N*3, *N*3 excites itself with an autaptic connection N3→N3 and inhibits *N*2 with an inhibitory connection N3→N2, thus putting the network in the *hA* state in which only the neuron *N*3 spikes continuously (Fig 3c-d). The continuous spiking of *N*3 also prevents the *Output* neuron from spiking (due to an inhibitory connection N3→Output). When signal A is followed by B, the inhibitory connection (B→N3) forces *N*3 (which maintains the state *hA*) to stop spiking, and the network goes into a quiescent state *hAB*. The input B is connected to *N*2 with a weak excitatory connection. This connection prevents an incorrect response of the network to a repeated signal B in the correct pattern ABC. The weight of this connection is adjusted so that the first B cannot activate the *Switch* neuron due to the continued inhibition of *N*3 (N3→N2). However, when *N*3 is released by the first B in the correct order, the second B can activate the *Switch* neuron, which in turn activates the neuron *N*3. As a result, the network goes back into the *Start* state (continuous spiking of both neurons *N*3 and *N*2). If the network receives signal C while in *hAB* state, the positive connection from the input C to *N*1 triggers several spikes in the neuron *N*1, and *N*1 passes the activity to the *Output* neuron. Consequently, the *Output* spikes in response to the last correct input if the lock has been released by the second-to-last correct signal. *N*1 also activates the *N*2/*Switch* neuron, which in turn activates the *N*3/*Lock* neuron, thus transforming the network back into the *Start* state.

**Fig 4:**
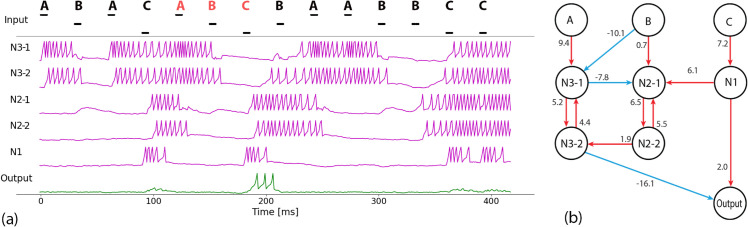
(a) Network activity of autapse-free network. (b) Autapse-free network created by replacing N3 and N2 autaptic neurons in the three-signal network (see Fig 3 ) with mutual pairs of excitatory neurons.

To show that the functional role of an autapse could be reproduced by a non-autaptic network, we handcrafted a network by replacing each autaptic neuron in a three-signal network with a mutually excitatory pair of neurons (Fig 4). Neuron *N*3 was replaced by two neurons, N3-1 and N3-2, mutually exciting each other with the same strength as the original autapse. Similarly, *N*2 was split into mutually exciting pair N2-1 and N2-2. Neuron N3-1 inhibits N2-1, whereas N2-2 excites N3-2. With a few evolutionary runs on the connection weights (fewer than 20), the handcrafted network yielded a perfect network with optimised connection weights (Fig 4). When the non-autaptic network receives the first correct signal A, it goes into *hA* state maintained by mutually exciting *N*3 pair (N3-1 and N3-2), N3-1 inhibits N2-1, whereas N3-2 locks the output neuron from spiking with a strong inhibitory connection. When signal A is followed by B, input B shuts down N3-1 with a strong inhibitory connection, thus deactivating the mutually exciting *N*3 pair, which releases the lock from the *Output* neuron. The network transitions to the *hAB* state, which is retained by no activity in the network. If the network receives C while in *hAB* state, *N*1 spikes several times and passes the activity to the *Output* neuron, causing it to spike for the last correct signal. *N*1 also activate the switch neuron to transform the network back into the *start* state, maintained by mutual excitation of *N*3 and *N*2 pairs.

### Performance of the handcrafted network for patterns of six signals

**Fig 5:**
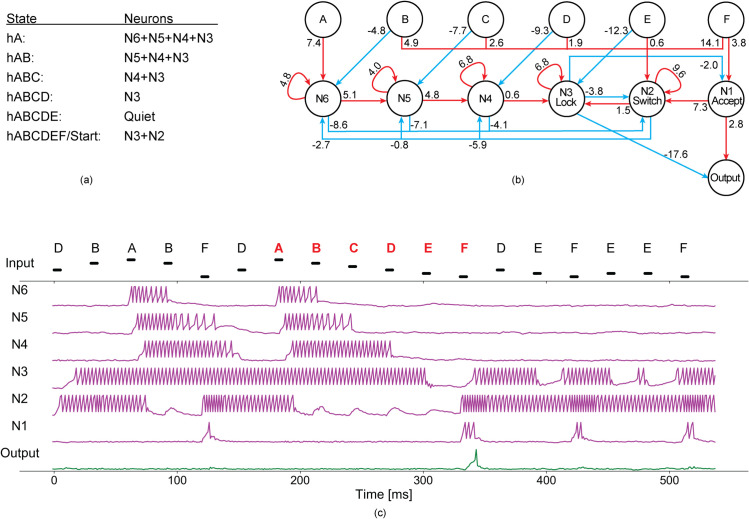
(a) The network states with corresponding active neurons. (b) The handcrafted network for recognising a pattern of length six. (c) The behaviour of each neuron in the network when a random stream of input signals A, B, C, D, E, and F is received. The different input levels represent input being received from different input channels; the voltage remains the same for all inputs.

The analysis of a network handcrafted for recognising a pattern of six signals shows that the network accomplishes the task of recognising the pattern with seven well-defined network states (Fig 5a). When the network receives the first correct signal A around 180 ms (Fig 5c), the network goes into the *hA* state represented by four active neurons, *N*6, *N*5, *N*4, and *N*3. The input A is connected directly to *N*6 through a strong excitatory connection, which causes *N*6 to spike on the arrival of A (Fig 5b). The excitatory autapse on *N*6 makes it spike continuously. This spiking activity, in turn, activates *N*5 and prevents *N*2 (the *Switch* neuron) from spiking. The spiking activity of *N*5 persists (due to the excitatory autapse), and *N*5 passes the activity to *N*4, which in turn activates *N*3 (the *Lock* neuron) in a similar way (Fig 5b-c). Meanwhile, when a signal B is received, the inhibitory connection from the input B to *N*6 shuts down *N*6, transforming the network into the state *hAB*, which is characterised by continuous spiking of *N*5, *N*4 and *N*3. The input B also excites the *Switch* neuron with a precise connection weight such that only a second B signal can activate the *Switch* neuron and transform the network back to the *Start* state. Similarly, suppose that AB is followed by the third signal C in the correct order. In that case, the inhibitory connection from the input C to *N*5 shuts down *N*5, transforming the network into the state *hABC*, represented by the continuous spiking of neurons *N*4 and *N*3. Next, if the network receives a input signal D in the correct order, it switches off *N*4 through an inhibitory connection from D to *N*4, transforming the network into the state *hABCD*, maintained by continuous spiking of *N*3 (the *Lock* neuron) only (Fig 5b-c). These four states from *hA* to *hABCD* are actively maintained by persistent spiking of four, three, two, and one neuron(s), respectively. It is important to note that *N*3 (the *Lock* neuron) is always active, except when the network receives the second-to-last signal E in the correct order, allowing the *Output* neuron to spike for the correct last signal F. The strong inhibitory connection from *N*3 to the *Output* neuron prevents *Output* from spiking for incorrect patterns. If the network is in the state *hABCD* (represented by continuous spiking of the *Lock* neuron) and it receives the second to the last signal E in the correct order, it switches to a quiescent state by releasing the lock from *Output* through an inhibitory connection from E to the *Lock* neuron (*N*3), which enables the *Output* neuron to spike in response to the last correct signal F (Fig 5b-c). The input F activates *N*1 through a positive connection, which in turn passes the activity to both the *Output* and the *Switch* neurons. The *Output* spikes in response to receiving the correct pattern ABCDEF, and the *Switch* neuron activates the *Lock* neuron. This transforms the network back into the *Start* state, where the *Switch* (*N*2) and the *Lock* neuron (*N*3) spike continuously.

To show that autapses are required for maintaining network states, we removed autapses one by one from an already evolved six-signal network and evaluated performance across all input combinations with an extended 50 ms silent interval. In every case, removing a single autapse resulted in degradation of the network consistent with the loss of the autaptic connection. For example, when the *N*6 autapse was removed, the network started responding to ACDEF pattern because the *hA* state (maintained by continuous spiking of *N*6,*N*5,*N*4, and *N*3 neurons) relaxed into state *hAB* (maintained by continous spiking of *N*5,*N*4, and *N*3) without waiting for the B signal. Similarly, the removal of *N*5, *N*4 or *N*3 autapses caused the output neuron to fire incorrectly for ABDEF, ABCEF and ABCDF, respectively. However, when the *N*2 autapse was removed, the output neuron spiked for almost all EFs because the start state (maintained by continuous spiking of *N*2 and *N*3) could no longer be activated, leaving the output neuron unlocked for spiking. This shows that each autaptic connection is required for maintaining a network states and preventing a network state from relaxing prematurely to the next.

**Fig 6:**
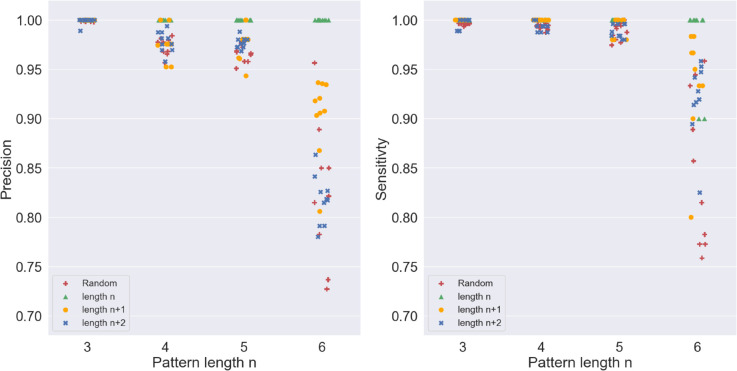
Performance degradation of handcrafted networks with increasing pattern length. The precision (left) and sensitivity (right) of the top 10 networks for each pattern size (3 to 6) are evaluated for a random sequence of length 1 million and all possible patterns of length n,n+1, and *n* + 2.

Finally, we evaluated the performance of the handcrafted networks in terms of precision and sensitivity. Precision is defined as the fraction of *Output* spikes that are correct, that is, the number of true positives divided by the total number of times the *Output* spiked, while sensitivity is the fraction of correctly classified target patterns, that is, the number of true positives divided by the actual number of correct patterns in the sequence. The top 10 networks for recognising patterns of lengths three, four, five, and six were re-evaluated for a random sequence of one million signals (Fig 6). It can be observed that the performance of the networks degraded with increasing length of the pattern. Our results showed that the precision (sensitivity) of the top 10 performing networks for pattern length six (ABCDEF) was between 0.73 (0.75) and 0.96 (0.96), whereas for the length five (ABCDE) and below, it consistently exceeded 0.94 (0.97). To investigate the slight degradation in performance with increasing signal length, the length-six networks were then tested with all possible permutations of six signals in six positions (length *n*) with replacement ${\;}^{6}P_{6}$ from AAAAAA to FFFFFF, six signals in seven positions (length n+1) with replacement ${\;}^{6}P_{7}$ from AAAAAAA to FFFFFFF, and six signals in eight positions (length n+2) with replacement ${\;}^{6}P_{8}$ from AAAAAAAA to FFFFFFFF (Fig 6). In a similar way, the top 10 performing networks for each pattern length *n* (three, four, and five) were tested with all possible permutations of length n,n+1, and n+2. For length five and below, the precision (sensitivity) consistently remained above 0.94 (0.97) (Fig 6). The testing of the networks with all possible patterns of lengths n,n+1, and *n* + 2 showed the impact of the proceeding signals (history) on the performance of the networks.

The top 10 perfect networks for each pattern length *n* (three four five and six) accurately responded to all possible permutations of length *n* (no history) with a precision and sensitivity of 1, except for two length-6 networks with a sensitivity of 0.90. To demonstrate that all false positives were caused by history or noise, all possible permutations of length six with two preceding signals (${\;}^{6}P_{8}$ from AAAAAAAA to FFFFFFFF) were presented to the top-performing length-six networks 10 times. The networks responded to the correct pattern XXABCDEF at least eight out of 10 times and responded at most four out of 10 times sporadically for a very small number of false positives. This clear margin indicated that handcrafted networks could perfectly recognise correct patterns up to six signals.

## Discussion

In the present study, we have explored how spiking neural networks can perform temporal pattern recognition tasks without updating or selecting conduction delays [19,27,30,32]. Understanding how the brain processes temporal information is not only an important research topic in computational neuroscience, but also crucial for reproducing artificial networks with brain-like computational capabilities [70]. The neural circuitry of the brain performs a large number of computational tasks, including signal processing, memory, classification, decision-making, etc. It is important to link functional behaviours to the corresponding neural connectivity of the brain [46,71,72]. However, the diverse set of computational capabilities, the enormous number of neurons, and the complexity of the brain make a principled mapping of neural connectivity to computational function difficult. In order to make inroads towards a basic understanding of information processing in neural systems, we evolved minimal artificial spiking neural networks for the computational task of temporal pattern recognition.

We found a clear association between structural connectivity and the functional properties of neural networks. We observed that the complexity of the networks increased with the length of the temporal patterns that could be recognised. The number of possible temporal patterns increased exponentially with the length of the patterns, making network training more difficult. Intuitively, the recognition of longer patterns required a larger number of neurons in the network, exponentially increasing the number of connections in the network. Larger and more interconnected networks were harder to train due to the computational cost and larger search space. Consequently, the artificial evolution could not produce an optimal minimal network for recognising a pattern of length four and above, and we resorted to handcrafting the topology of networks for longer patterns (and using artificial evolution only to optimise the weights in these networks).

One of the most salient observations is the emergence of autapses in our spiking neural networks. Although autapses are widely observed in the brain, their functional role in neural information processing is not yet fully clear. Autapses have previously been shown to play a significant role in memory maintenance [73], oscillatory activity of a single neuron [74], switching between different network states [75], synchronisation of network activity [43], and signal detection [76]. Here, we show the importance of excitatory autaptic connections for network state transitions and network state maintenance. In our work, all active network states are maintained by excitatory autaptic connections, whereas state transitions occur when an excitatory autaptic connection of an active neuron is silenced by a strong inhibitory connection. Furthermore, we examined the relationship between the number of excitatory autapses in the network and the length of temporal patterns that can be recognised. The number of autapses in a network constrains and is equal to the number of distinct actively maintained states in the network. We observed that a minimal network recognising a pattern of length *n* requires at least *n*–1 autaptic connections. This is a necessary condition for a network to be able to maintain network states. A sufficient condition is that the weights of the autaptic connections are strong enough to keep the spiking activity from dying out in the presence of longer silent intervals.

Redundant connections in a network have a potential role in learning temporal sequences [77–79]. Although the aim of the present work was to obtain minimal networks for recognising patterns, some redundancy is essential in the network. For example, the extended network that recognises a pattern of length four requires two redundant connections (Fig 2c). If we remove input A and neuron *N*4, the four-signal network is reduced to a three-signal network with two redundant connections; an excitatory connection from input D to the *Switch* (*N*2) neuron and an inhibitory connection from the *Lock* (*N*3) to the *Accept* (*N*1) neuron. The connection from input D to *N*2 is required in a four-signal network to activate the switch neuron as soon as the network receives the last correct signal, while the redundant inhibitory connection from *N*3 to *N*1 prevents the *Output* neuron from spiking when the last signal D is received in a wrong order. Although the *Output* neuron is prevented from spiking directly by the *Lock* (*N*3) neuron, this connection reduces the activity that reaches the *Output* neuron when the lock is active.

Moreover, the obtained networks prevented repeated signals by precise excitatory connections from the inputs to the *Switch* neuron *N*2. For example, in a three-signal network (Fig 3) the input B has a weak excitatory connection to *N*2, which prevents a repeated signal B. The weight of this connection is adjusted so that the first B cannot activate the *Switch* neuron due to the continued inhibition of N3(N3→N2). However, when *N*3 is released by the first B in the correct order, the second B can activate the *Switch* neuron, which in turn activates the neuron *N*3. As a result, the network goes back into the *Start* state. When the connection between input B and *N*2 was removed, the network began to respond to the pattern $AB^{+}C$. A similar pattern was observed in the six-signal network (Fig 5). When the excitatory connection from input B to *N*2 was removed, the network began responding to $AB^{+}CDEF$. Similarly, when excitatory connections form inputs C, D and E to *N*2 were removed one at a time (with previously removed connections restored before testing the next), the network responded to $ABC^{+}DEF$, $ABCD^{+}EF$ and $ABCDE^{+}F$, respectively. When all excitatory connections from inputs B, C, D, and E to *N*2 were removed, the network responded to $AB^{+}C^{+}D^{+}E^{+}F$. Since the obtained networks could be mapped onto a finite-state automata, they successfully recognised repeated signals after a small adjustment, i.e., after removal of the excitatory connections from the inputs to the *N*2 neuron). However, non-consecutive repetitions (e.g. ABA) or palindromes (e.g. ABCBA) cannot be captured by the current finite state network, as they would require a pushdown automaton. Extending the network to recognise context-free patterns is a potential direction for future work.

Information processing in neural systems is affected by the presence of noise. Noise in the nervous system has previously been documented to play a computational role [80–85]. We previously showed [69] that networks evolved in the presence of noise are robust to intrinsic (perturbation of parameters) and extrinsic (variation of silent intervals) disturbances. In fact, noise enables networks to maintain network states (a form of memory) when the silent interval between signals is increased during evolution. One of the key findings is that the introduction of noise during evolution simplifies the networks instead of further complicating them. The resulting networks are robust to the removal of connections. Thus, we could prune excessive connections without impairing the performance of the networks. As a result, the simplified (evolved and pruned) networks are more computationally efficient and easier to understand. In contrast, the networks that evolved in the absence of noise were fragile and a slight variation of neuronal parameters or network topology would completely alter the functionality of these networks [69].

The approach of handcrafting the network topology provides insight into neural information processing and the interplay between network connectivity and functional behaviour. However, this work has several limitations that could be addressed in future studies. In particular, the maximum number of active neurons maintaining a network state increases with an increasing length of the patterns that can be recognised. As the number of connections in the network grows, their weights become more difficult to optimise. Due to this limitation, further scaling of the topology produces suboptimal networks. One possible solution to this problem is to build a larger network by connecting two or more three-signal (perfect) networks to recognise more extended patterns. However, the approach of hierarchically connecting smaller networks to build a larger network is not straightforward. We have investigated several ways to interconnect smaller networks to recognise more extended patterns of length 5 and 6, but none of them could outperform the sequential extension of the topologies presented in Fig 2. In future studies, it would be interesting to explore other possible topologies where the maximum number of neurons that can have sustained activity (neurons with autapses), represent a network state that is invariant to the pattern length.

An AdEx neuron can generate a wide range of spiking behaviours to a step current, depending on the choice of initial parameters [57]. In this study, we are using the parameters for the simplest type of spiking behaviour, that is, tonic or regular spiking [57]. A standard leaky integrate-and-fire model (LIF) can also generate this behaviour in response to a step current. In the scope of this study, we do not take advantage of the rich spiking behaviours that an AdEx neuron can have. The neurons in our networks are limited to three possible states: active (tonic spiking), intermittently active, and nonactive (quiet). We observed that an activated neuron may speed up or slow down after receiving an input signal, but we did not take these firing rate changes into account when analysing network behaviour. Identifying network states based on the spiking behaviour of a single neuron would require longer intervals between signals to notice the difference between their responses. Studying the state transition at the level of a single neuron in the network could result in more efficient solutions. For example, different neuronal behaviours, such as bursting, tonic, and irregular spiking, may represent distinct network states. Moreover, different spiking/bursting frequencies could also represent different network states.

Finally, in this study we use a genetic algorithm to optimise connection weights in a handcrafted topology. Since all connections in the handcrafted topology are defined according to the state transition table and both the topology and network states are known, a more systematic approach like backpropagation-through-time could be employed instead of the genetic algorithm to optimise the weights in the network. This could be done by (i) calculating the errors between the current and the desired network states and (ii) adjusting the connection weights to reach the desired states.

## Conclusions

The present study used a novel combination of evolving and handcrafting spiking neural networks to explore potential mechanisms for temporal pattern recognition in neural systems. Our work demonstrates a link between structural network connectivity and functional network behaviour. A systematic analysis of the resulting networks indicates that excitatory autaptic connections can play a key role in memory maintenance and network state transitions. We predict that the presence of autapses in a neural system implies that the system might be able to perform temporal pattern recognition. A systematic investigation of links between autaptic connections and temporal coding should be an exciting topic for future experimental work.
